# ABA-glucose ester hydrolyzing enzyme ATBG1 and PHYB antagonistically regulate stomatal development

**DOI:** 10.1371/journal.pone.0218605

**Published:** 2019-06-24

**Authors:** Jeffrey Allen, Konnie Guo, Dongxiu Zhang, Michaela Ince, Fabien Jammes

**Affiliations:** 1 Department of Biology and Program in Molecular Biology, Pomona College, Claremont, California, United States of America; 2 USDA-ARS, Systematic Mycology and Microbiology Laboratory, Beltsville, Maryland, United States of America; National Taiwan University, TAIWAN

## Abstract

The integration of conflicting signals in response to environmental constraints is essential to efficient plant growth and development. The light-dependent and the stress hormone abscisic acid (ABA)-dependent signaling pathways play opposite roles in many aspects of plant development. While these pathways have been extensively studied, the complex nature of their molecular dialogue is still obscure. When mobilized by the *Arabidopsis thaliana* β-glucosidase 1 (AtBG1), the glucose ester-conjugated inactive form of ABA has proven to be a source of the active hormone that is essential for the adaptation of the plant to water deficit, as evidenced by the impaired stomatal closure of *atbg1* mutants in response to water stress. In a suppressor screen designed to identify the molecular components of AtBG1-associated physiological and developmental mechanisms, we identified the mutation *variant of AtBG1 traits (vat1)*, a new mutant allele of the red light/far-red light photoreceptor *PHYTOCHROME B (PHYB)*. Our study reveals that *atbg1* plants harbor increased stomatal density in addition to impaired stomatal closure. We also provide evidence that the *vat1/phyb* mutation can restore the apparent transpiration of the *atbg1* mutant by decreasing stomatal aperture and restoring a stomatal density similar to wild-type plants. Expression of key regulators of stomatal development showed a crosstalk between AtBG1-mediated ABA signaling and PHYB-mediated stomatal development. We conclude that the AtBG1-dependent regulation of ABA homeostasis and the PHYB-mediated light signaling pathways act antagonistically in the control of stomatal development.

## Introduction

A particular change in a plant’s environment often has similar effects on both the development of the stomata and the stomatal aperture [1–3]. For example, red light exerts a positive influence on guard cell development and induces stomatal opening, in particular through activation of the photoreceptor PHYB [4–7]. Stomatal development in *Arabidopsis thaliana* begins when a protodermal cell differentiates via a multi-step process that produces three successive guard cell precursors. The differentiation is under the control of a number of basic helix-loop-helix (bHLH) transcription factors [3, 8, 9]. Five bHLH genes in *Arabidopsis* regulate stomatal development and the entry of protodermal cells into stomatal lineages. Three of these, *SPEECHLESS (SPCH)*, *MUTE*, and *FAMA*, are members of subfamily Ia and act in sequence to mediate cellular transitions during stomatal development. The other two are *SCREAM (SCRM/ICE1)* and *SCRM2*, members of subfamily IIIb, which act redundantly to coordinate the action of SPCH, MUTE, and FAMA via heterodimerization. Protodermal cells differentiate into meristemoid mother cells via molecular mechanisms that are not yet fully understood, although it is known that SPCH is required for this transition. Once differentiated, a meristemoid mother cell divides to produce a meristemoid and a stomatal lineage guard cell under the control of SPCH. The meristemoid then differentiates into a guard mother cell under the control of MUTE. The guard mother cell subsequently differentiates into guard cells, a process controlled by FAMA, and produces mature stomata [10]. Several regulators of these transcription factors have been identified. The photoreceptor PHYB acts as a positive regulator of stomatal development partly by negatively regulating the photomorphogenic regulator COP1, an indirect repressor of SPCH, MUTE, and FAMA activity [5]. Recent evidence also implicates the PHYTOCHROME INTERACTING FACTORS 4 (PHYB-PIF4) module in the direct regulation of SPCH for the control of stomatal development [4, 11].

The importance of abscisic acid (ABA) in stomatal aperture has long been recognized and its role in stomatal development is increasingly being revealed. Abscisic acid inhibits entry of cells into stomatal lineages, thereby reducing stomatal densities on leaves [12]; it is of interest that in this, ABA mirrors its role in limiting water loss via stomatal closure, and opposes the role of light in the promotion of both stomatal development and opening [13]. The ABA-deficient mutant *aba2-2* has more stomata than the wild-type plant, and this phenotype can be rescued with exogenous ABA. Also of interest, it has been noted that fewer meristemoid mother cells were produced by osmotically-stressed plants due to a MAPK-mediated decrease in SPCH protein [14].

Levels of ABA are regulated by a combination of de novo synthesis, catabolism, transport, and conjugation [15]. The *A**rabidopsis* *t**haliana* β-glucosidases 1 and 2 (AtBG1 and AtBG2) can both cleave inactive ABA-glucose ester conjugate to its active free form in a one-step conversion that constitutes a rapid mechanism of regulation of stomatal aperture [16, 17]. In contrast, de novo synthesis, while important, proceeds via a 10-step pathway. The consequences of the loss-of-function of AtBG1 and AtBG2 are additive, but the phenotype of the *atbg1* T-DNA mutant is much more disrupted than that of *bg2* mutants [17]. The *atbg1* mutant plant has lower concentrations of active ABA than wild-type (WT) plants, which accounts for its impaired stomatal closure and increased water loss under drought stress. The *atbg1* mutants also has marked developmental defects such as yellowing and serration of the leaves, suggesting that AtBG1 is not only involved in the rapid adaptation of the plant to stress, but also in the regulation of ABA-dependent developmental processes [16]. However, the regulation of AtBG1 and AtBG2 in ABA homeostasis and adaptation to water stress remains largely unknown.

We wished to identify molecular components of the signaling cascade that regulates ABA-related developmental events and the role of AtBG1 in the adaptation of *Arabidopsis thaliana* plants to abiotic stresses. To this end, we screened an ethyl methanesulfonate (EMS)-mutagenized population of *atbg1* mutant plants and isolated a point mutation that appeared to restore the drought tolerance of the *atbg1* mutant. This point mutation, which we named *variant of atbg1 traits 1* (*vat1)* was mapped as a new allele of *PHYB* and is responsible for a phenotype consistent with an inability of the plant to respond to red light. Further phenotypic analyses of the single mutants and *atbg1/vat1(phyb*) double mutants showed that the increased drought sensitivity of *atbg1* mutants was due both to impaired stomatal closure and more stomata. We observed that introducing the *vat1(phyb)* mutation into *atbg1* plants limited light-induced stomatal opening and restored the number of stomata to a level similar to that of WT plants, contributing to the restored drought tolerance of the *atbg1/vat1(phyb)* double mutants. Finally, from expression analysis of regulators of stomatal development, we conclude that AtBG1 and PHYB have opposing roles, particularly in the regulation of SPCH and MUTE, and act antagonistically in the regulation of stomatal development.

## Materials and methods

### Plant material and growth conditions

*Arabidopsis thaliana* seeds of ecotypes Landsberg erecta (Ler) or Columbia (Col-0) were supplied by the Arabidopsis Biological Resource Center. Seeds were incubated at 4°C for 2 days to break dormancy prior to germination. After incubation, seeds were surface-sterilized and plated under sterile conditions on medium containing Murashige and Skoog basal salt mixture supplemented with 0.05% MES, 1% sucrose, 0.8% of phytoagar (PhytoTechnology Laboratories, Lenexa, KS, USA). Plants were routinely grown on Metro-Mix 360 (Grace-Sierra, Milpitas, CA, USA) soil in controlled growth chambers at 22°C, and supplied with 12 h of light with a photon flux density between 50 to 120 μmol photons·m^-2^·sec^-1^ (light intensity varied in different experiments), followed by 12 h darkness.

### Genetic mapping of *vat1(phyb)* mutation

A population of M2 seeds of *atbg1* mutants mutagenized with 0.2% (w/v) EMS (Sigma-Aldrich, St Louis, MO, USA) was produced on a Col-0 background, as described by Jander et al. [18]. Mutagenized plants that harbored the *vat1(phyb)* phenotype, detected by the characteristically elongated organs, were crossed with Col-0 plants and then self-crossed. The absence of the characteristic phenotype in the F1 progeny and segregation analysis of the F2 plants indicated that the *vat1*(*phyb*) mutation was recessive and in a single locus. A series of five crosses of EMS-treated plants with the characteristic phenotype with Col-0 plants, combined with self-crossings, allowed us to isolate the *vat1*(*phyb*) mutation from other EMS mutations.

For map-based cloning, *vat1(phyb)* plants of the Col-0 ecotype were crossed with plants of the Ler ecotype. The progeny was then self-crossed to generate the F2 plants. The F2 plants with the characteristic elongated organs phenotype was used for the mapping. PCR-based genetic markers were selected to analyze the DNAs for the initial linkage test. Forty different DNA samples were used together with 31 markers equally distributed into the Arabidopsis genome [19]. The recombination frequency was calculated according to the formula from Sanchez-Serrano and Salinas [20]: F = (no. heterozygotes + 2 x no. homozygous Ler)/(2 x no. plants). Once the linkage between the mutation and the chromosome II had been established, 12 markers were used with 40 individual F2 plants. This first step of the mapping showed that the PLS2 marker co-segregated with the mutation. Additional tests performed on 160 plants allowed us to locate the mutation between the markers PLS2 and PLS6. Analysis of the gene composition in the genome region combined with the particular light-dependent phenotype of *vat1(phy)b* plants led to the sequencing of the PHYB gene. The primers used for the mapping of the mutation can be found in S1A and S1B Table.

### Water loss assay

Three leaves were detached from plants (three plants for each line) in growth chambers (under normal growth conditions: 22°C, 30% relative humidity) and weighed at various times after detachment. The transpiration rate of plants was measured with thermal imaging, using a T450sc infrared camera (FLIR, Nashua, NH, USA). Leaves of similar size were detached from plants that had six to eight leaves, placed in a growth chamber (22°C, 50 μmol photons·m^-2^·sec^-1^, high ventilation, 30% humidity), and the change in leaf surface temperature was measured over 10 minutes. Thermal images were analyzed using the FLIR ResearchIR Software.

### Determination of chlorophyll content

Chlorophyll content was determined as described by Gechev et al. [21]. Leaf samples (50 mg fresh weight) were taken in triplicate from mature plants, placed in 1 mL of 80% acetone, and incubated overnight at 4°C. Spectrometric measurements of the supernatant were made in 1-mL glass cuvettes. Pigment contents were calculated according to the Beer-Lambert-Bouguer law, using the extinction coefficients, as previously described [21, 22].

### Stomatal movement assay

Stomatal movement assays were performed with 3-week-old plants as previously described [23]. Epidermal peels were incubated in 20 mM KCl, 10 mM MES-Tris pH 5.6 for 2.5 hours under light and then treated with 10 μM ABA for 30 min. Stomatal apertures (width/length) were evaluated using a light microscope before and after ABA treatment.

### Hypocotyl length, stomatal density and index

Stomatal density, index and hypocotyl length were measured using scanning electron microscopy. For stomatal density and index, fully developed cotyledons were dissected from plants with at least four adult leaves. Primary imprints of the adaxial surface of the cotyledons were made using high-resolution dental resin (Ted Pella, Redding, CA, USA). Positives were made using PELCO epoxy resin (Ted Pella, Redding, CA, USA) and coated with gold before observation on an Hitachi SU-70 scanning electron microscope. Alternatively, samples were dehydrated in ethanol and critical point dried (EMS 3000; Electron Microscopy Sciences) prior to gold sputter coating. Samples were imaged on an Hitachi SU3500S at Rancho Santa Ana Botanic Garden. Images were analyzed using the ImageJ Software [24]. For hypocotyl length, a similar method was applied to one-week-old plants.

### RNA purification and RT-qPCR

Plant materials (10–50 mg fresh weight) was collected and immediately frozen in liquid nitrogen. Samples were rapidly transferred to a lysing matrix-A tube (Millipore, Burlington, MA, USA) and homogenized with the FastPrep-24 homogenizer (Millipore, Burlington, MA, USA) for 40 s at 6 m·s^-1^. RNA was extracted with the GenElute Universal Total RNA Purification Kit (Sigma-Aldrich, St Louis, MO, USA), following the manufacturer’s instructions and using an On-Column DNAse treatment and the On-Column DNAse I digestion set (Sigma-Aldrich, St Louis, MO, USA). Eluted RNA was quantified with an ND-1000 spectrophotometer (NanoDrop Technologies, Wilmington, DE, USA) and first-strand cDNAs were synthesized with the iScript cDNA synthesis kit (Biorad, Hercules, CA, USA) according to the manufacturer’s instructions. Quantitative PCR results were generated using the 2^-ΔΔCt^ method [25]; TUBULIN BETA-9 CHAIN gene (TUB9, at4g20890) was used as the reference gene. Three independent biological replicates for each line were performed. Significance tests were done using pair-wise t-tests. Graphs and statistical analyses were generated using Prism (GraphPad, La Jolla, CA). Details regarding the genes tested and their primers are provided in S2 and S3 Tables.

### Sequence comparison and structural analysis

Sequences were collected from the Arabidopsis Information Resource and the National Center for Biotechnology Information. Multiple sequence alignments were done with ClustalOmega [26] and Boxshade v3.21 [27]. PHYB structure (PDB: 4OUR)[28] was analyzed using the SWISS-MODEL software [29] and the Discovery Studio Visualizer 2.5.5 (Accelrys Software, Inc.).

## Results

### *vat1* restores drought tolerance of the *atbg1* mutant

**Fig 1:**
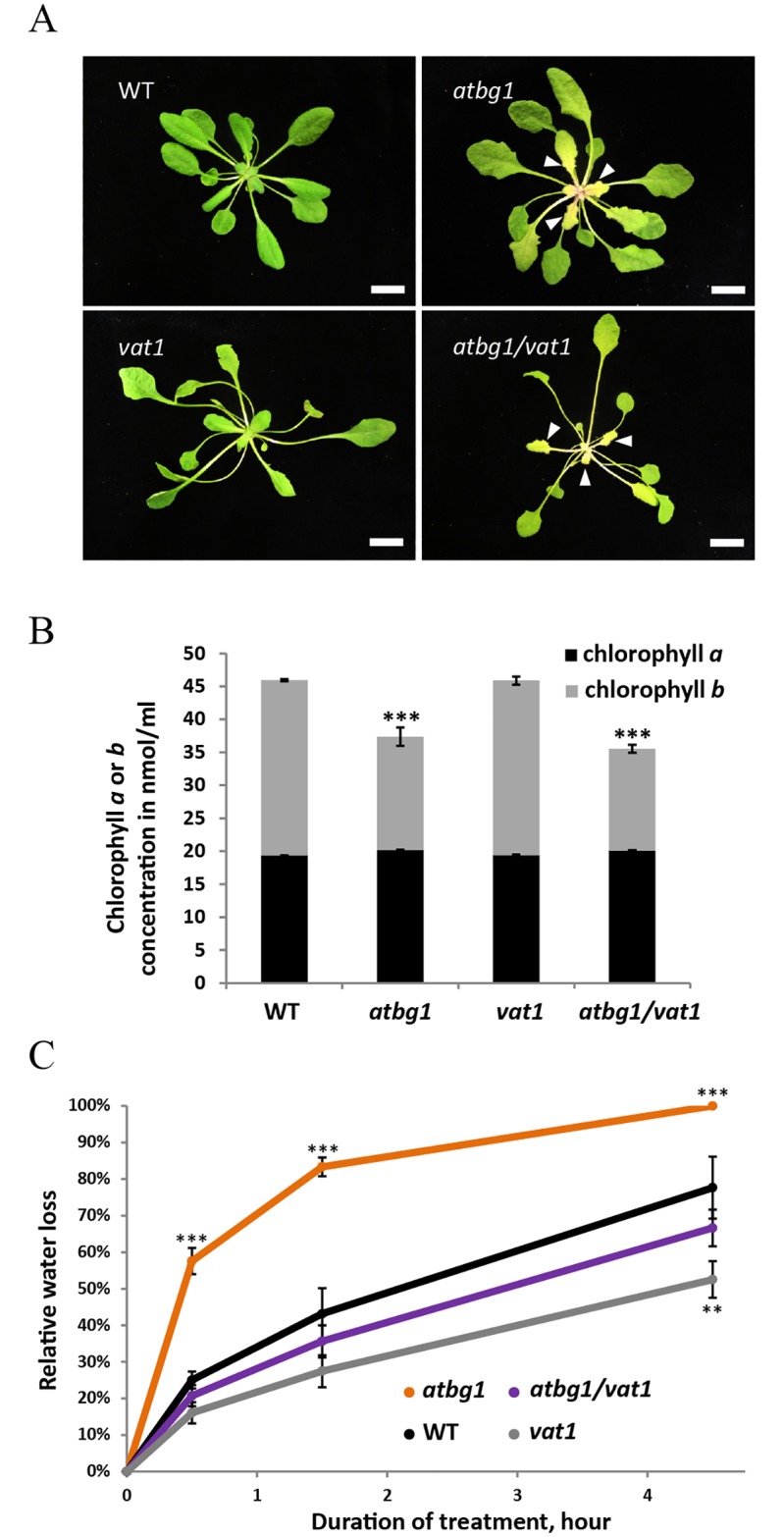
Developmental and physiological defects in *atbg1*, *vat1* and *atbg1/vat1* mutants compared to WT. (A) Adult *atbg1* plants display a yellow leaf phenotype, more pronounced in emerging leaves. *vat1* petioles and hypocotyl appear longer compared to WT plants. The leaves of *atbg1/vat1* double mutants show the additive effects of the developmental defects observed in each single mutant. Arrows indicate pale green to yellow leaves in *atbg1* and *atbg1/vat1* mutants. Bar = 1cm. (B) Chlorophyll *a* and *b* contents. A decreased concentration in chlorophyll *b* may contribute to the pale green to yellow color of the aerial organs in *atbg1* mutants and *atbg1*/*vat1* double mutants. Error bars are SEM (n = 3).*** P <0.001 (*t*-test). (C) Leaf transpiration assay. Comparison of water loss rates shows greater water loss in *atbg1* mutants and restored water loss in *atbg1/vat1* double mutant as compared to WT. Error bars are SEM (n = 3).***, P<0.001 (*t*-test); **, P<0.05 (*t*-test).

Developmental differences between *atbg1* plants and an EMS-mutagenized *atbg1* population were used as primary cues to identify suppressors of the physiological consequences of the *atbg1* mutation. Among the candidates identified, one corresponded to an independent and recessive allele in a single locus and was named “*v*ariant of *atbg1* traits *1” (vat1)*. The *atbg1/vat1* double mutants had significantly longer organs than either the *atbg1* or WT plants (Fig 1A). The isolation and characterization of homozygous *vat1* single mutant plants indicated that the mutation in the *VAT1* locus was solely responsible for the elongated organs phenotype, and that it did not depend on an *atbg1* genotypic background (Fig 1A). As previously observed in *atbg1* mutants, the *atbg1/vat1* double mutants had a yellow-leaf phenotype associated with a decrease in chlorophyll b (Fig 1B). Therefore, we concluded that the combined effects of the *vat1* mutation and the *atbg1* mutation resulted in elongated organs and yellow leaves, respectively. To test whether the *vat1* mutation conferred a modified tolerance of *atbg1* plants to water stress, plants that had been grown under optimal water availability and light were challenged with water stress. Detached leaves were weighed over a 4-h period to assess the ability of plants to tolerate the stress by limiting transpiration (Fig 1C). As expected, the *atbg1* mutants had decreased tolerance to water stress compared to WT plants, due to their inability to mobilize the pool of glucose-conjugated ABA into active ABA (Fig 1C). The introduction of the *vat1* mutation in *atbg1* mutants, however, restored drought tolerance to a level similar to WT plants. In addition, the *vat1* single mutant plants showed a modest, but statistically significant, increase in tolerance to dehydration compared to WT plants.

### *vat1* is a new mutant allele in *PHYTOCHROME B*

**Fig 2:**
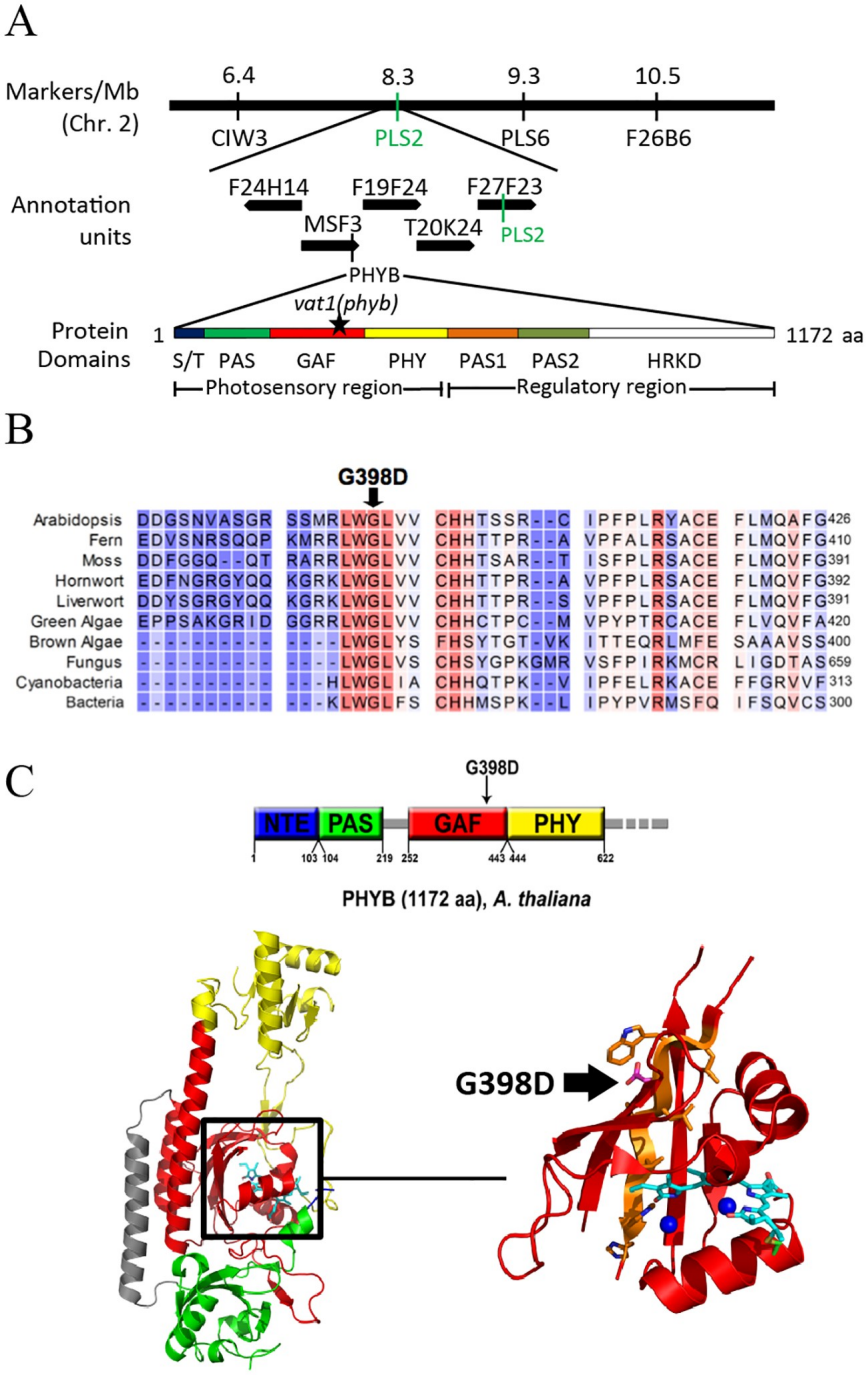
The *vat1* mutation is a new allele in the *PHYB* gene. (A) Molecular markers mapped the mutation in the genetic interval between markers CIW3 and PLS2 on Arabidopsis chromosome 2. The corresponding physical map from the Arabidopsis Genome Initiative presented *PHYB* as a promising target gene based on the preliminary observations of *vat1* mutant phenotype. The sequence of *PHYB* gene in the *vat1* mutant contains a G-to-A mutation resulting in the substitution of the predicted glycine residue at position 398 to aspartate (black star) in the GAF domain. (B) Sequence alignment of part of the *A*. *thaliana* PHYB GAF domain to homologous proteins from other organisms. The mutated glycine 398 is shown by a black arrow. Red color indicates highly conserved residues while blue indicates less conserved residues. GenBank accession numbers are: Arabidopsis, AEC06808.1; Fern, BAA33775.1; Moss, EDQ61588.1; Hornwort, AHX73747.1; Liverwort, 635149067; Green algae, BAL36428.1; Brown algae, AKN34536.1; Fungus, AAZ57422.1; Bacteria, CEI76617.1. (C) Top: The photosensory module of PHYB consists of 4 domains: NTE (blue), PAS (green), GAF (red) and PHY (yellow). Bottom: The photosensory module contains a bilin chromophore bound by a thioether linkage to C357 in the GAF domain. The chromophore (cyan) is stabilized by hydrogen bonds and van der Waals interactions to nearby residues in the GAF domain and two water molecules (blue spheres) (Burgie et al., 2014). The G398D *vat1(phyb)* (shown in magenta) is located within a highly conserved β-sheet (orange) near the chromophore.

The elongated phenotype of plants carrying the *vat1* mutation provided a practical way to identify the molecular basis for this defect by map-based cloning. Using PCR-based analysis of genetic markers on an F2 mapping plant population, the mutation *vat1* was mapped to a region between the molecular markers CIW3 and PLS2 on chromosome 2 (Fig 2A). Among the genes in this region, the gene encoding PHYB, the red/far-red light photoreceptor phytochrome B, drew our attention as it is an important player in the light-dependent inhibition of hypocotyl and petiole elongation. Subsequent DNA sequencing revealed a single nucleotide mutation (G-to-A) in the first exon of *PHYB*, which results in the change of amino acid residue 398 from Gly to Asp (G398D). The G398D mutation is located within the GAF domain (cGMP phosphodiesterase/adenylyl cyclase/FhlA) in the photosensory module of phytochrome B [30]. The GAF domain, to which the chromophore is covalently bound at residue 357, is flanked by the PER, ARNT, and SIM (PAS) and phytochrome-specific GAF-related (PHY) domains (Fig 2A). Multiple sequence alignment of photoreceptors from bacteria to higher plants showed that the Gly-398 residue is highly conserved and, to our knowledge, its role has not been documented (Fig 2B).

**Fig 3:**
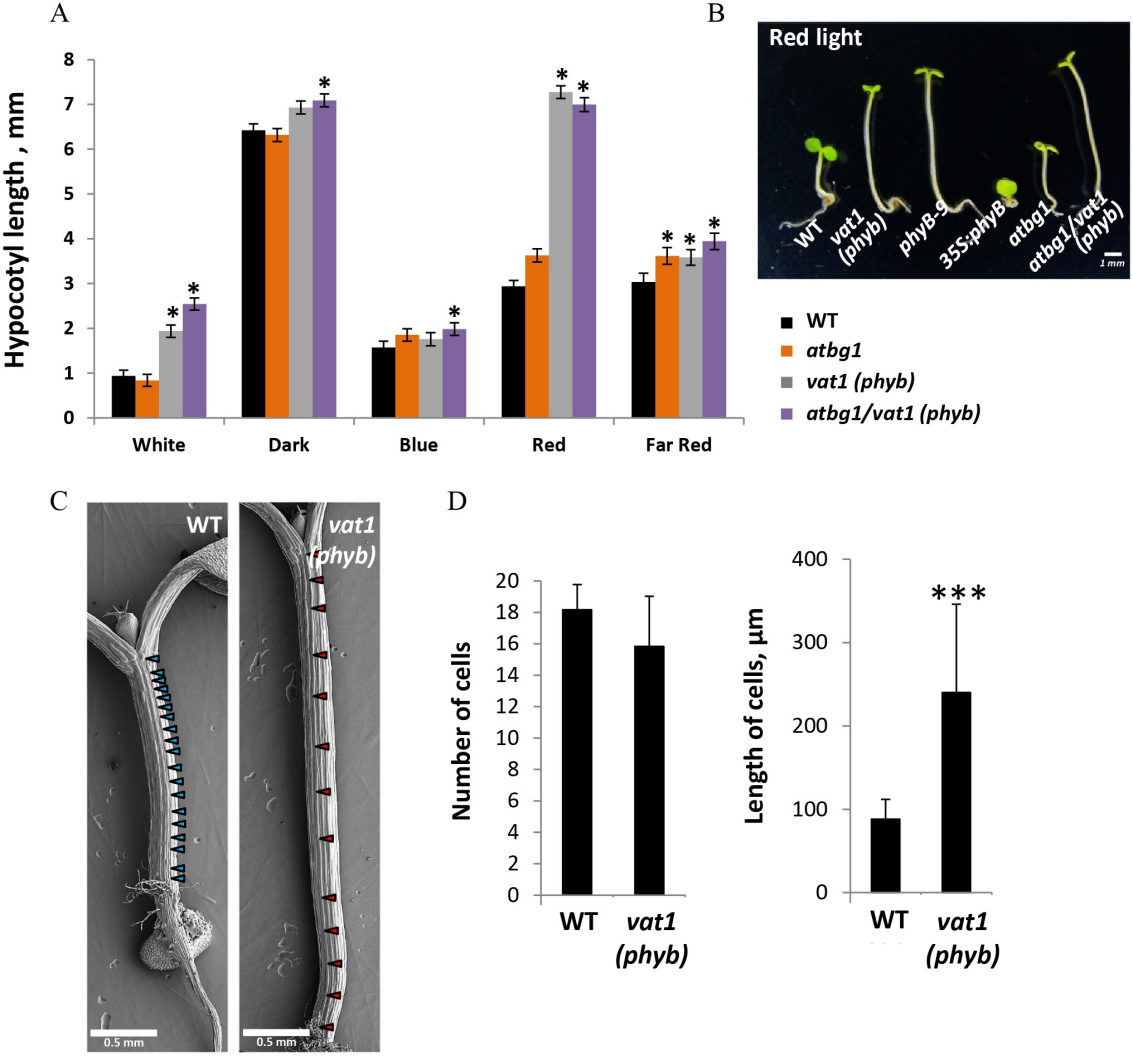
The phenotype of *vat1(phyb)* plants indicates a loss of function of the PHYB protein. (A) Left: Hypocotyl length measurements under different wavelengths show a significant red light-dependent increase in hypocotyl length in *vat1(phyb)* and *atbg1/vat1(phyb)* mutants compared to *atbg1* and WT plants. Error bars are SEM (n = 20).***, P <0.001 (*t*-test). (B) The response of *vat1(phyb)* mutant to red light is similar to the previously characterized loss-of-function mutant *phyB-9*. (C) SEM images of *Arabidopsis* hypocotyl epidermis show more elongated cells in *vat1(phyb)*. One-week-old seedlings were grown under constant light. Arrows indicate the junctions between cells. (D) Average (± SD) cell number and length measured in 3 rows of cells from 2 different seedlings. ***, *P*<0.001 (*t*-test).

Structural modeling of the N-terminal region of PHYB using the crystal structure of the photosensory module [28], showed that G398 is located inside the binding pocket of the chromophore, in the vicinity of amino acids that are important for the covalent binding of the chromophore, as well as for the structure or stability of the pocket (Fig 2C). Therefore, the *vat1(phyb)* mutation, resulting in the substitution of a small hydrophobic amino acid (Gly) with a polar hydrophilic amino acid (Asp), may destabilize the hydrophobic binding pocket by affecting its flexibility or intramolecular interactions. A characteristic feature of impaired PHYB function is the activation of skotomorphogenic development, such as cell elongation under light [31]. We measured hypocotyl lengths under light of different wavelengths and observed a predominantly red light-dependent effect on hypocotyl elongation in mutants harboring the *vat1*(*phyb*) mutation (Fig 3A). This absence of inhibition of hypocotyl elongation in response to red light in *vat1(phyb)* and *atbg1*/*vat1(phyb)* is similar to the well-characterized loss-of-function mutant *phyB-9* (Fig 3B). Scanning electron microscopy-assisted quantification of hypocotyl cell numbers and cell length showed that the increased hypocotyl length observed in the lines bearing the *vat1(phyb)* mutation was the consequence of a significant increase in cell elongation (Figs 3C and 3D). Altogether, these results support the idea that *vat1(phyb)* is a new mutant allele of PHYB with a severely impaired responsiveness to red light suggesting a loss-of-function of the red/far-red light photoreceptor.

### The increase in stomatal density in *atbg1* is reversed by the *vat1(phyb)* mutation

**Fig 4:**
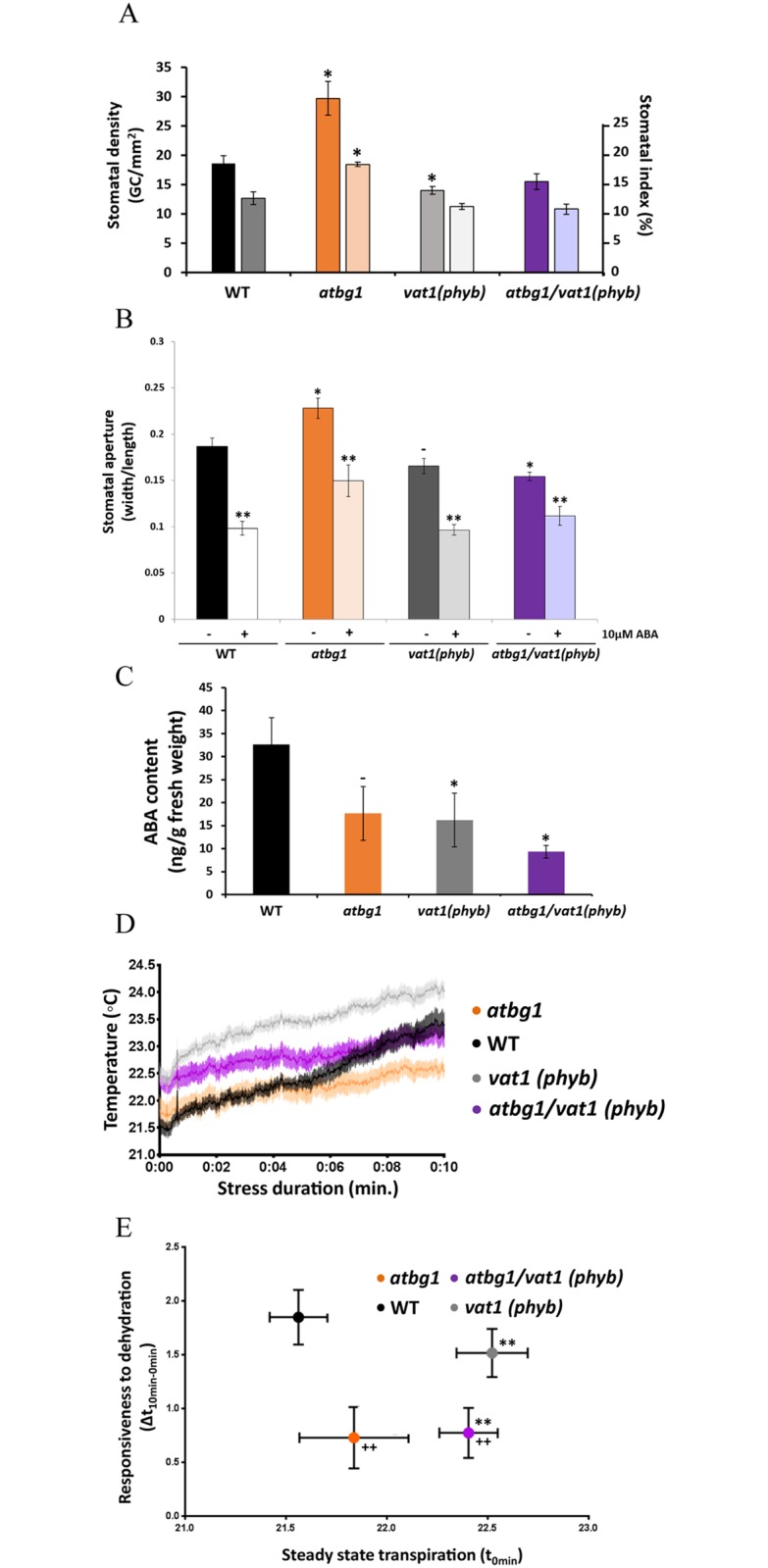
The *vat1(phyb)* mutation restores stomatal density, decreases the steady-state transpiration of *atbg1* mutant, but does not restore its ability to rapidly respond to water stress. (A) Stomatal density (left axis, dark bars) and index (right axis, light bars) of the four lines. Three fully expanded cotyledons from each line were imaged using scanning electron microscopy. *atbg1* mutants show a significantly increase in stomatal density and index (*t*-test, *P<0.05) that is restored to WT levels in *atbg1/vat1(phyb)* plants. Error bars represent standard error (n = 3). (B) Stomatal movement assay shows an increased aperture of *atbg1* stomata and a decreased stomatal aperture of *vat1(phyb)* single mutant and *atbg1/vat1(phyb)* double mutant (*t*-test, *P<0.05, ^-^P = 0.054). All lines are responsive to 10 μM ABA (**P<0.01). Error bars represent standard error (n = 40–60). (C) Quantification of free ABA. Well-watered plants from each line were used to isolate and quantify ABA by QTRAP LC-MS/MS (*t*-test, *P<0.05, ^-^P = 0.1). Error bars represent standard error (n = 3). (D) Average change in leaf surface temperature between t = 0 and t = 10 minutes following detachment from the plant. Shaded regions represent the standard error (n = 9). (E) Responsiveness to dehydration *versus* steady-state transpiration. The initial temperature (t_0min_) of a leaf is an indicator of its steady-state transpiration, or initial stomatal conductance, while the change in leaf temperature over a period of intense water stress (Δt_10min-0min_) indicates the ability of the plant to close its stomata. Error bars are standard error (n = 9). ** indicates significantly different values of steady-state transpiration as compared to WT (*t*-test, P<0.01). ++ indicates significantly different values of responsiveness to dehydration as compared to WT (*t*-test, P<0.01).

Stomatal conductance is dependent on stomatal aperture and stomatal number. We wished to investigate the mechanism by which drought tolerance is restored in the *atbg1*/*vat1(phyb)* double mutant. To this end, we measured stomatal density and index in the *atbg1* and *vat1(phyb)* single mutants, the *atbg1/vat1(phyb)* double mutant, and in WT plants. Stomatal formation in Arabidopsis leaves follows a tip-to-base gradient that complicates studies of the timing of stomatal development. With a reduced or absent tip-to-base gradient, cotyledons constitute a good model to study stomatal development [32]. Fully expanded mature cotyledons from plants grown under low irradiance (50 μmol photons.m^-2^.s^-1^) were imaged with electron microscopy, and the stomatal density and index were determined (Fig 4A). The *atbg1* mutant had significantly higher stomatal density and index than WT plants, while the stomatal density and index of *atbg1*/*vat1(phyb)* mutants were similar to WT plants (Fig 4A). Although light is a major positive regulator of stomatal development, the presence of a normal stomatal index in a loss-of-function *phyb* mutant grown under low irradiance has previously been documented by Casson et al. (2009). Similarly, the *vat1(phyb)* single mutant, despite a moderately lower stomatal density, did not show a significant difference in stomatal index either, compared to control. Our observation of increased stomatal density and index in *atbg1* may be evidence that the impaired drought tolerance of the mutant in response to water stress is the consequence of an abnormally high number of stomata in addition to the previously reported impaired ability for stomata to properly close in response to water stress. These results also indicate that the *vat1(phyb)* mutation restored stomatal density to levels similar to WT plants in *atbg1*/*vat1(phyb)* mutants, thereby establishing a relationship between PHYB-mediated light signaling, AtBG1-dependent control of ABA homeostasis, and the regulation of stomatal development.

### The drought tolerance of *atbg1*/*vat1(phyb)* is not due to restored responsiveness of stomata to water stress

Plant tolerance to water stress is a multi-factorial trait that depends, in addition to stomatal density, on stomatal aperture. In that regard, the most influential factors are light and ABA. Stomatal movement assays indicated that the stomatal aperture of *vat1(phyB)* was slightly decreased compared to WT plants, an expected result considering the positive role of red light in the promotion of stomatal opening [7], and this observation was more pronounced in the *atbg1/vat1(phyb)* double mutant (Fig 4B, no ABA treatment). The stomata of *atbg1* plants however showed an increased aperture compared to WT plants. This could be due to the previously reported lower basal levels of ABA in this mutant [16]. All lines were responsive to the exogenous application of 10 μM ABA (Fig 4B, after ABA treatment). Interestingly, before ABA treatment, the decreased stomatal aperture of *atbg1/vat1(phyb)* double mutant, similar to that of *vat1(phyb)* single mutant, suggest that, for the control of stomatal opening, red light signaling may supplant the function of AtBG1. Quantification of ABA levels in well-watered plants confirmed decreased ABA levels in *atbg1* single mutant (Fig 4C). In addition, a decreased level of ABA was observed in the *vat1(phyb)* mutant and an additive effect between the *atbg1* mutation and *vat1(phyb)* mutation was noted in the double mutant. Taken together, our study of stomatal movements and ABA levels suggest that the *vat1(phyb)* mutation might contribute to the restoration of the drought tolerance of *atbg1* mutant by decreasing its light-dependent stomatal aperture in the absence of stress rather than increasing ABA sensitivity and/or restoring free ABA concentrations.

To verify our conclusions, we measured the stomatal conductance under non-stressed conditions as well as the ability to close the stomata in leaves responding to water stress. To that end, *atbg1* and *vat1(phyb)* single mutants and *atbg1/vat1(phyb)* double mutants were submitted to an acute water stress by removing leaves from the plants and placing them under constant air flow for 10 min. Evaporation of water through stomata at the leaf surface, a measure of stomatal conductance, was assessed with infrared thermal imaging (Fig 4D) [33]. The surface temperature immediately after leaf removal, before the leaf could perceive the stress and respond with rapid stomatal closure, provides information about the steady-state stomatal conductance. This parameter was not significantly different in WT plants and *atbg1* mutants despite a trend suggesting moderately more opened stomata in *atbg1* mutants. This indicates that despite its foliar developmental defects, a balanced stomatal conductance can be preserved in the *atbg1* mutant in the absence of stress (Fig 4D and 4E). Conversely, the steady-state foliar temperature of both *vat1(phyb)* and *atbg1*/*vat1(phyb)* was significantly higher than WT plants and *atbg1* mutants. That is indicative of a reduced transpiration under normal conditions, prior to the perception of the water stress and likely due to the inability of these plants to perceive red light.

Stomatal responsiveness was calculated from the difference in surface temperature of dissected leaves under intense water stress over 10 min according to the equations: Δt = t_10min_ − t_0min_, in which t is foliar temperature. We observed significantly lower Δt in the *atbg1* mutants and the *atbg1*/*vat1* double mutants compared to WT plants, indicating that they both had similar impaired ability to close their stomata in response to water stress. The stomatal response of the *vat1(phyb)* single mutant was not significantly different than that of WT plants (Fig 4E). Taken together, these results indicate that the improved drought tolerance of *atbg1*/*vat1(phyb)* compared to *atbg1* is not because of a restored ability to close the stomata in response to the stress, but rather due to a difference in steady-state transpiration.

### The *vat1(phyb)* mutation counteracts the up-regulation of stomatal development regulators in *atbg1*mutants

**Fig 5:**
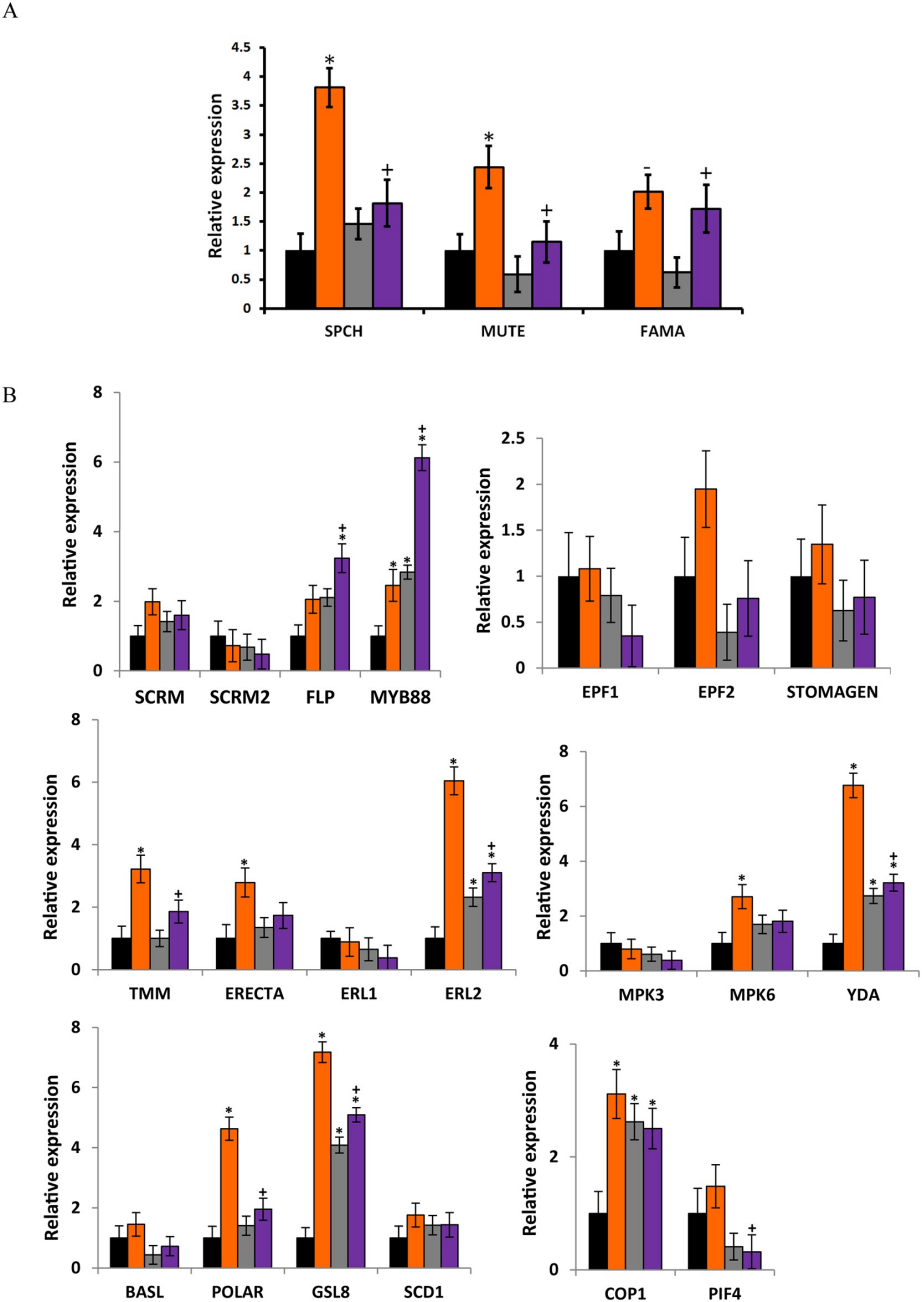
The increase in stomatal density in *atbg1* mutants correlates with the overexpression of key regulators of stomatal development. (A) Relative expression levels of genes involved in guard cell differentiation. The transcription factors *SPCH* and *MUTE* are highly up-regulated in the *atbg1* mutants but to a lesser extent in *atbg/vat1(phyb)* mutants. Expression levels were normalized to *TUB9* using the 2^-ΔΔCt^ method from three biological replicates. Error bars show standard error (n = 3). * indicates significantly different gene expression as compared to WT (P<0.05). ^+^ indicates significant differential gene expression levels in *atbg1* or *vat1(phyb*) single mutants as compared to the *atbg1/vat1(phyb)* double mutant (*t*-test, P<0.05). ^-^, P = 0.06 (*t*-test). (B) Relative expression levels of genes involved in stomatal development in five-day-old cotyledons. Expression levels were normalized to the reference gene TUB9 using the 2^-ΔΔCt^ method from three biological replicates. Error bars are standard errors. * indicates significantly different gene expression as compared to WT (*t*-test, P<0.05). ^+^indicates significantly different gene expression levels in *atbg1* or *vat1(phyb*) single mutants as compared to the *atbg1/vat1(phyb)* double mutant (*t*-test, P<0.05).

We next wished to determine the molecular factors responsible for increased stomatal density in *atbg1*. This was undertaken by analyzing the expression of 22 genes involved in stomatal development (S3 Table). The differences in stomatal density between lines can introduce a quantitative bias in the study of the expression of genes involved in guard cell formation. To avoid this issue, we measured by RT-qPCR the expression of these genes in young (5-day-old) seedlings, in which the emerging cotyledons were not yet showing statistically significant differences in stomatal density and only minor differences in stomatal index (S1 Fig). This approach is commonly used in developmental biology and aims at focusing on the earliest molecular events responsible for a phenotype while avoiding quantitative biases. Fig 5A focuses on the genes encoding three key transcription factors involved in promoting guard cell differentiation: *SPCH*, *MUTE*, and *FAMA*, which were up-regulated 3.8-fold, 2.5-fold, and 2-fold, respectively, in the *atbg1* mutant compared to the WT plants. Expression of *SPCH* and *MUTE* was lower in *vat1(phyb)* mutants and *atbg1*/*vat1(phyb)* double mutants compared to *atbg1*, and similar to that observed in WT plants (expression ratios were between 1 and 1.8). Interestingly, while *FAMA* was up-regulated in *atbg1*, its expression levels didn’t seem affected by the *vat1(phyb)* mutation as indicated in the *atbg1*/*vat1(phyb)* double mutants.

**Fig 6:**
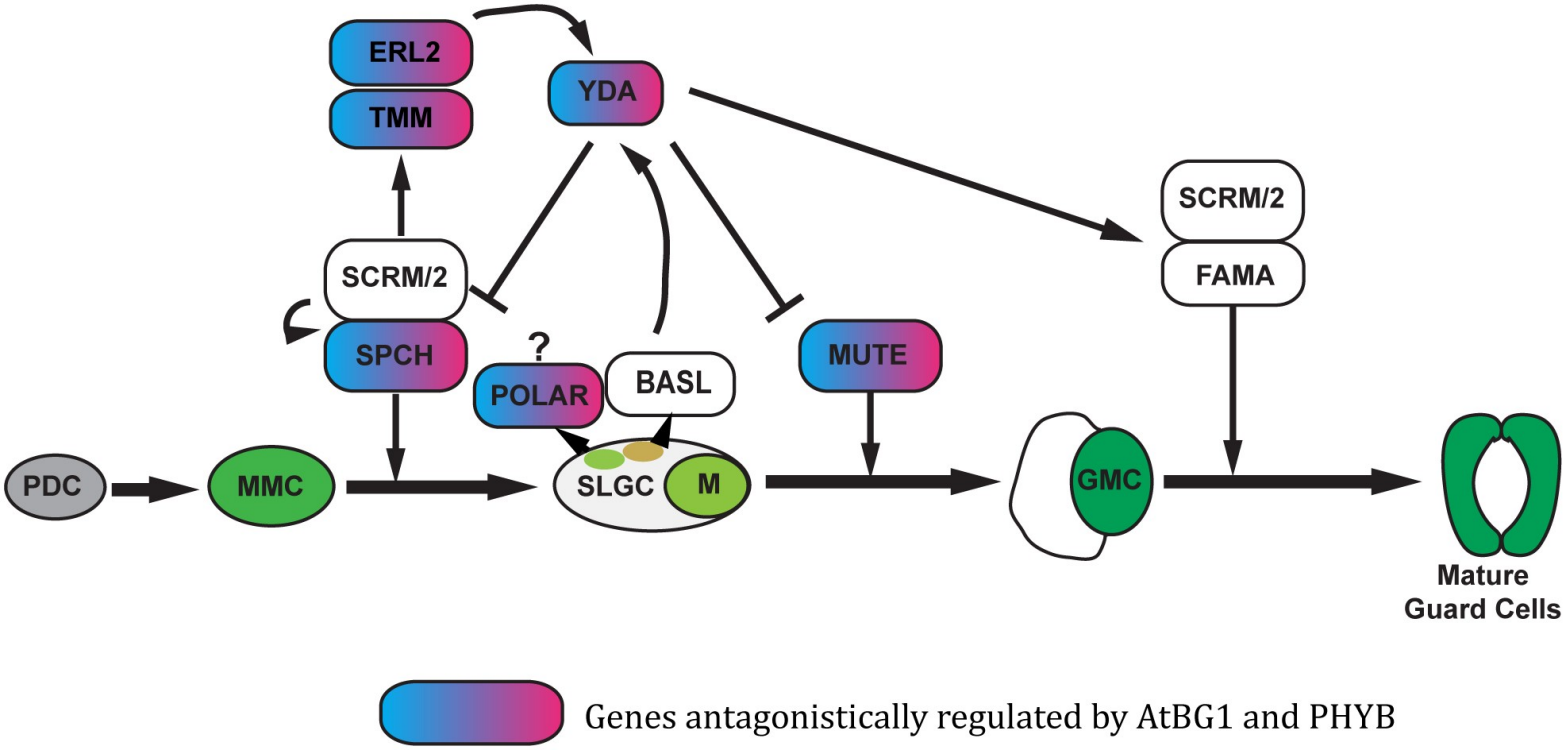
Influence of AtBG1 and PHYB on the expression of genes involved in stomatal development. This model is based on Han and Torii [34]. Only the genes showing antagonistic influences of AtBG1 and PHYB when comparing the *atbg1/vat1(phyb)* double mutant to either *atbg1* or *vat1(phyb*) single mutant are shown.

The differential expression of 19 additional genes involved in the spacing and patterning of stomata was analyzed (Fig 5B). Two genes, *YDA* and *ERL2*, were highly up-regulated; both had a 6-fold increase in expression level in *atbg1* mutant plants compared to WT plants. Both genes were also up-regulated in *vat1(phyb)* and *atbg1*/*vat1(phyb)* plants, but to a lesser extent. This observation illustrates the complexity of the crosstalk between AtBG1- and PHYB-dependent pathways and that the unique molecular and physiological landscape of the *atbg1*/*vat1(phyb)* double mutant is a valuable tool to better understand how plants balance the integration of ABA and red light signals. In addition, while *TMM* was clearly up-regulated in *atbg1* mutants (≥2-fold), the up-regulation was more modest in the *atbg1*/*vat1(phyb)* double mutants (Fig 5B). Similarly, the expression of *POLAR*, a gene associated with cellular asymmetry of meristemoids, was more up-regulated in the *atbg1* mutants (4.5-fold) than in the *atbg1*/*vat1(phyb)* mutant (2-fold). In conclusion, the increased stomatal density in *atbg1* is associated with up-regulation of several key genes involved in stomatal development, and the restoration of a normal stomatal density in the *atbg1*/*vat1(phyb)* double mutants is associated with either a less drastic over-expression of these genes, or with levels of expression similar to that of WT plants (Fig 6).

## Discussion

### AtBG1 regulates drought tolerance by modulating stomatal aperture and stomatal density

Land plants frequently encounter water stress and have evolved mechanisms to cope with it [35]. One such mechanism is the rapid closure of stomata, induced by the phytohormone ABA, which prevents evapotranspiration [13, 36]. Abscisic acid-induced stomatal closure, however, comes at a price: in addition to limiting water loss, influx of atmospheric CO_2_ through the stomata and carbon fixation is reduced [37, 38]. When considering molecular mechanisms opposing ABA to maintain the energetic balance and metabolic homeostasis, light-regulated pathways, which are responsible for the promotion of stomatal opening and photosynthesis, come to mind even though light-independent pathways leading to stomatal opening have also been identified [23, 39]. While ABA and light signaling pathways have been the subject of numerous studies [40], their complexity has greatly limited our understanding of the molecular dialogues between them. Here, we report on our investigation of the role of AtBG1, a β-glucosidase capable of hydrolyzing the inactive ABA-glucose conjugate to active ABA, and the red light/far-red light photoreceptor PHYB in the adaptation to water stress.

In this study, we observed that, in addition to controlling stomatal aperture [16], AtBG1 negatively regulated stomatal development. The *atbg1* mutant has significantly greater stomatal density, which is accompanied by up-regulation of several genes that regulate stomatal development (Figs 4A and 5). To our knowledge, the increased stomatal density of the *atbg1* mutant has not yet been reported, and is consistent with the findings that ABA inhibits cell entry into stomatal lineage [12]. While the ABA biosynthesis pathway is intact in the *atbg1* mutant, its growth and ABA-mediated responses are more disrupted than has been observed for ABA-deficient mutants such as *aba2* [12, 16]. We suggest, based on these results, that AtBG1 ensures normal stomatal density through its negative regulation of stomatal development, in addition to permitting rapid formation of active ABA for adaptation to water deficit.

The role of ABA in the response to drought has been well studied, but an examination of the integration of ABA signaling in stomatal development has just begun [1, 34]. It is known that drought and osmotic stress reduce the number of meristemoid mother cells and inhibit stomatal development [14]. The over-expression of genes that regulate stomatal development in *atbg1* mutants (Fig 5), such as *SPCH* and *MUTE*, is consistent with previous findings that ABA acts upstream of *SPCH* to negatively regulate stomatal development [12]. In addition, the over-expression of *SPCH* in *atbg1* mutants is likely responsible for the over-expression of *TMM*, *ERL2*, and *POLAR* which are downstream targets of *SPCH* [41] and certainly play a role in the increased stomatal density in *atbg1* mutants. While our study supports the notion that *AtBG1* negatively regulates *SPCH*, the mechanism remains to be determined.

### *vat1(phyB)* restores the drought tolerance of *atbg1* by restoring expression of stomatal development genes

In addition to its central role in plant photosynthesis, light influences plant physiology and development in many ways. Specifically, the red/far-red light photoreceptor PHYB has received considerable attention in the light-mediated regulation of plant development and physiology [31, 42, 43]. In this investigation we sought to characterize the novel PHYB mutant *vat1(phyb)* and determine how this mutation restores the drought tolerance of the *atbg1* mutant. The *vat1(phyb)* mutation is a new G398D mutation located within the GAF domain of the photosensory module; it likely impairs the sensing of red light. The nearest photochemically characterized mutation to the G398D is the V401S mutation [28], which severely impairs PHYB. The mutation results in a protein unable to undergo thermal reversion to the Pr state [28]. In regard to *vat1(phyb)*, we note that the mutation severely alters the physiological function of PHYB, but the effect of the mutation at the intramolecular level is uncertain. One hypothesis is that the G398D mutation could prevent the photochemical conversion of the Pr state to the Pfr. Alternatively, the G398D mutation could increase the rate of thermal reversion in such a way that there would be very little Pfr state at any given time. A photochemical analysis performed as described by Burgie et al. [28] would help discriminate between these two possibilities.

We showed that *vat1(phyb)* mutants were more drought-tolerant than WT plants and that the *vat1(phyb)* mutation restored the drought tolerance of *atbg1* mutants in the *atbg1/vat1(phyb)* double mutant plants (Fig 1). Interestingly, the mutation did not seem to restore the drought tolerance of *atbg1* by restoring the ability of the stomata to respond to stress. Our analysis of the initial transpiration rate in the different lines, their adaptation to water stress, and stomatal density provides an explanation for the restoration of drought tolerance in *atbg1/vat1(phyb)* plants compared to *atbg1* plants. First, by limiting the ability of the plant to respond to red light, the mutation *vat1(phyb)* prevented *atbg1* plants from fully opening their stomata under normal conditions, making the *atbg1/vat1(phyb)* plants less prone to water loss. Second, we showed that drought sensitivity of *atbg1* was also due to increased stomatal density and that the inactivation of PHYB-dependent pathways could suppress this phenotype.

While Kang et al. [5] demonstrated the roles of PHYs, CRYs, and COP1 in stomatal development, the role of PHYB is by no means solved. The elucidated pathway suggests that, in response to light, PHYB regulates stomatal development by negatively regulating COP1, YDA, and the MAPK cascade, removing the repression of SPCH, MUTE, and FAMA [5]. Interestingly, our results do not show a significant reduction in stomatal density or in the expression of *SPCH* in *vat1(phyb)* single mutants compared to WT plants. This is likely a consequence of growing the plants under low irradiance to minimize the broad physiological impact of impaired red light signaling. Indeed, it is known that high irradiance exaggerates the effect of defective PHYB and reveals some macroscopic defects, such as impaired stomatal development [4]. It is important to note, however, that the absence of stomatal development defects in loss-of-function PHYB mutants at low irradiance does not necessarily preclude a molecular role of the photoreceptor in stomatal development under these conditions, but rather suggests that other mechanisms are sufficient to visibly maintain the physiological balance.

In our study, some of the stomatal development targets of PHYB-dependent pathways were, indeed, revealed in the *atbg1/vat1(phyb)* phenotypic landscape rather than in the *vat1(phyb)* single mutant, showing that the double mutant is a valuable tool for exploring the role of PHYB in the regulation of stomatal development at low irradiance. Our results do not show an increase in COP1 following the loss of PHYB as described previously [5], but PHYB likely acts elsewhere and certainly via interactions with PIF4 [3, 4, 44]. PIF4 is a bHLH transcription factor, as are the crucial cell fate transcription factors SPCH, MUTE, FAMA [45]. The latter three of these bHLH transcription factors physically interact with the more distantly related bHLH transcription factors SCRM and SCRM2 to form heterodimers [46]. This heterodimerization is necessary for the activity of the core transcription factors. It has been suggested that, as a bHLH transcription factor, PIF4 may influence stomatal development by interacting with the core members of the stomatal development pathway, modifying the activity of these proteins and thus influencing stomatal development [4]. The work of Casson et al. [4] suggested that PIF4 promotes, in a PHYB-dependent manner, stomatal development in response to light signals. In other environmental conditions, however, PIF4 has been shown to be a core component of high-temperature signaling that can suppress SPCH and lead to decreased stomatal density in warmer temperatures [11]. The conclusion by Casson et al. [4] that PIF4 positively regulates red light mediated stomatal development in a photoactivated-PHYB-dependent manner is in agreement with our observation that PIF4 was slightly down-regulated in both the *vat1(phyb)* (*P* = 0.06) and *atbg1/vat1(phyb)* mutants (*P* = 0.047; Fig 5B). Interestingly, an increased expression of *PIF4* in response to light has been reported by Huq and Quail [44], but the equal efficiency of both red light and far-red light on that matter suggested only a partial role for PHYB in the regulation of *PIF4* expression. Overall, while post-transcriptional modifications play an evident role in ABA and red/far-red light signaling, our results support the conclusion that PHYB contributes to stomatal development by counteracting the negative influence of AtBG1-dependent mechanisms on the expression of key regulators, such as *SPCH* and *MUTE*. The hypothesis that PIF4 may reinforce the role of the photoreceptor in that manner deserves further investigation.

## Conclusion

We conclude that the ABA-glucose hydrolyzing enzyme AtBG1 negatively regulates stomatal development. We have identified *vat1(phyb)*, which is a new mutant allele of the red/far-red light photoreceptor PHYB. The characterization of single mutants *atbg1* and *vat1(phyb)*, and the double mutant *atbg1/vat1(phyb)* permits us to conclude that AtBG1 and PHYB antagonistically control key regulators of stomatal development.

## Supporting information

S1 File Supplementary materials and methods. Quantification of ABA.
(DOCX)

S1 Fig Stomatal density and index in the four lines in five-day-old cotyledons. Stomatal density: With 0.1<pvalue<0.4, no statistically significant difference between the lines is detected. Error bars represent the standard error (n = 3). Stomatal index: Only the *atbg1/vat1(phyb)* double mutant shows a very modest but statistically significant difference (*P = 0.03) compared to WT. Error bars represent the standard error (n = 3).
(DOCX)

S1 Table Primers used for the mapping. (DOCX)

S2 Table List of qPCR primers. (DOCX)

S3 Table Stomatal development genes tested by RT-qPCR. (DOCX)
