# Supplementary material for: ABA-glucose ester hydrolyzing enzyme ATBG1 and PHYB antagonistically regulate stomatal development
Source: PLoS One. 2019 Jun 24;14(6):e0218605. doi: 10.1371/journal.pone.0218605 (PMC6590796; doi:10.1371/journal.pone.0218605)
Supplement: S1 File — Quantification of ABA. (DOCX) [file pone.0218605.s001.docx]

Quantification of ABA

Materials

Analytical reference standards for the analytes (+/­-)-abscisic acid (ABA; Sigma-Aldrich, St. Louis, MO) as well as the internal standard d_6_-ABA (ICON Isotopes, Dexter, MI) were used. LC-MS grade ethanol (MeOH) and acetonitrile (ACN) were sourced from J.T. Baker (Avantor Performance Materials, Radnor, PA) and LC-MS grade water was purchased from Honeywell Research Chemicals (Mexico City, Mexico). Stock solutions were prepared in 50% methanol and stored at -80° C. Standard solutions were prepared fresh in 30% methanol.

Phytohormone Extraction

Abscisic acid was extracted at a tissue concentration of 100 mg/mL in ice cold 1:1 MeOH:ACN containing a mixture of stable-isotope labeled standards (d_6_-ABA) at a concentration of 2.5 μM. The samples were homogenized with a TissueLyzer-II (Qiagen) for 5 minutes at 15 Hz then centrifuged at 16,000 g for 5 minutes at 4^o^ C. The supernatants were transferred to new 2 mL tubes and the pellets were re-extracted with 1:1 ice cold MeOH: ACN without the internal standards. The extracts were combined and dried in a vacuum centrifuge. The samples were reconstituted at a concentration of 500 mg/mL (based upon the original sample mass) in 30% methanol, centrifuged to remove particulates then passed through a 0.8 μm polyethersulfone spin filter (Sartorius, Goettingen, Germany) prior to dispensing into HPLC vials or well plates for LC-MS/MS analysis.

LC-MS/MS Analysis

ABA was quantified using a targeted multiple reaction monitoring (MRM)/isotope dilution-based LC-MS/MS method. Two microliters of the reconstituted samples were loaded onto a 1.0 x 100 mm 1.7 μm particle size Acquity C_18_-BEH column (Waters, Milford, MA) attached to an ekspert microLC200 (eksigent Technologies, Dublin, CA) and HTC xT-PAL autosampler (CTC Analytics, Zwingen, Switzerland) running 0.1% acetic acid in water (A) and 0.1% acetic acid in 3:1 ACN:MeOH (B) as mobile phases. The phytohormones were eluted using a linear gradient from 20% B to 95% B over ten minutes at a flow rate of 15 μL per minute and detected online with a 6500 QTRAP (SCIEX, Framingham, MA) equipped with a Turbo-V Spray ion source (SCIEX, Framingham, MA). Source parameters were set as follows: curtain gas, 25 psi; source gas1, 50 psi; source gas2, 50 psi; collisionally activated dissociation (CAD) gas set to ‘high’; interface heater temperature, 550˚ C; ionspray voltage set to ±4500. Individual analyte and internal standard ions were monitored using previously optimized MRM settings programmed into a polarity switching method (cytokinins and auxins detected in positive ion mode, others detected in negative ion mode). Analyst 1.6.2 software (SCIEX, Framingham, MA) was used for data acquisition; MultiQuant 3.0.2 software (SCIEX, Framingham, MA) was used for data analysis. The detected phytohormones were quantified based upon comparison of the analyte-to-internal standard integrated area ratios with a standard curve constructed using those same analytes, internal standards and internal standard concentrations (25 μM d_5_-dinor-OPDA and ^13^C_6_^15^N-JA-Ile; others 2.5 μM). The analytical method was calibrated over the linear range of 64 fmol to 10 pmol loaded on column.
