# Supplementary material for: ABA-glucose ester hydrolyzing enzyme ATBG1 and PHYB antagonistically regulate stomatal development
Source: PLoS One. 2019 Jun 24;14(6):e0218605. doi: 10.1371/journal.pone.0218605 (PMC6590796; doi:10.1371/journal.pone.0218605)
Supplement: S1 Fig — Stomatal density: With 0.1<pvalue<0.4, no statistically significant difference between the lines is detected. Error bars represent the standard error (n = 3). Stomatal index: Only the atbg1/vat1(phyb) double mutant shows a very modest but statistically significant difference (*P = 0.03) compared to WT. Error bars represent the standard error (n = 3). (DOCX) [file pone.0218605.s002.docx]

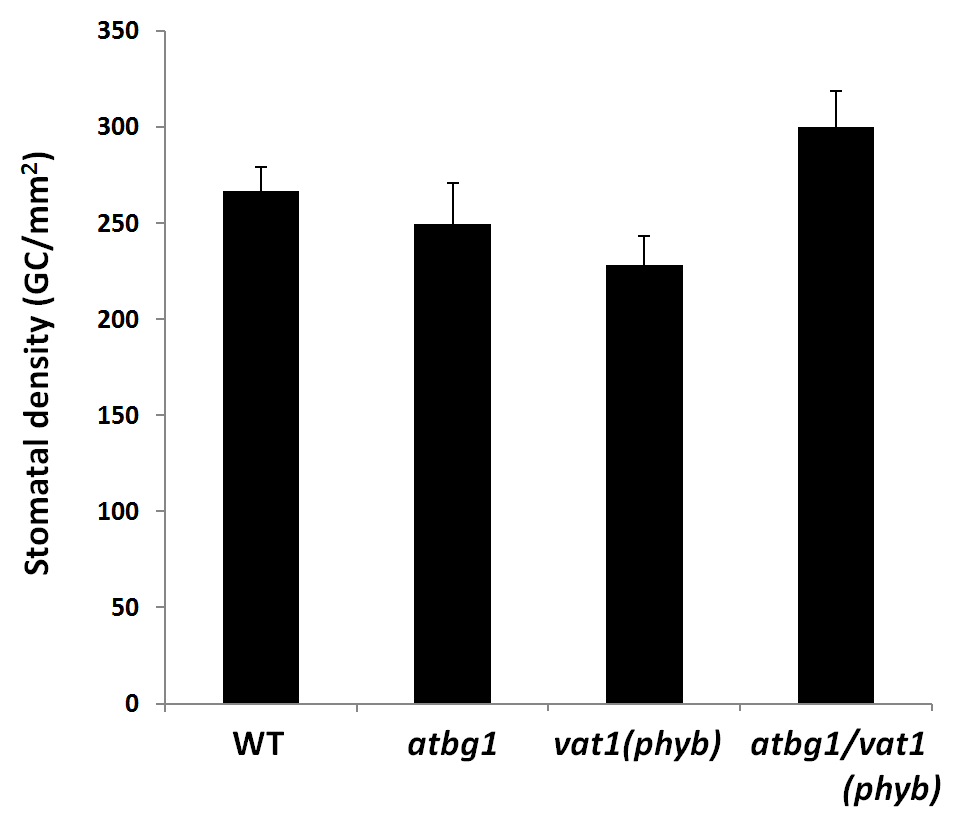


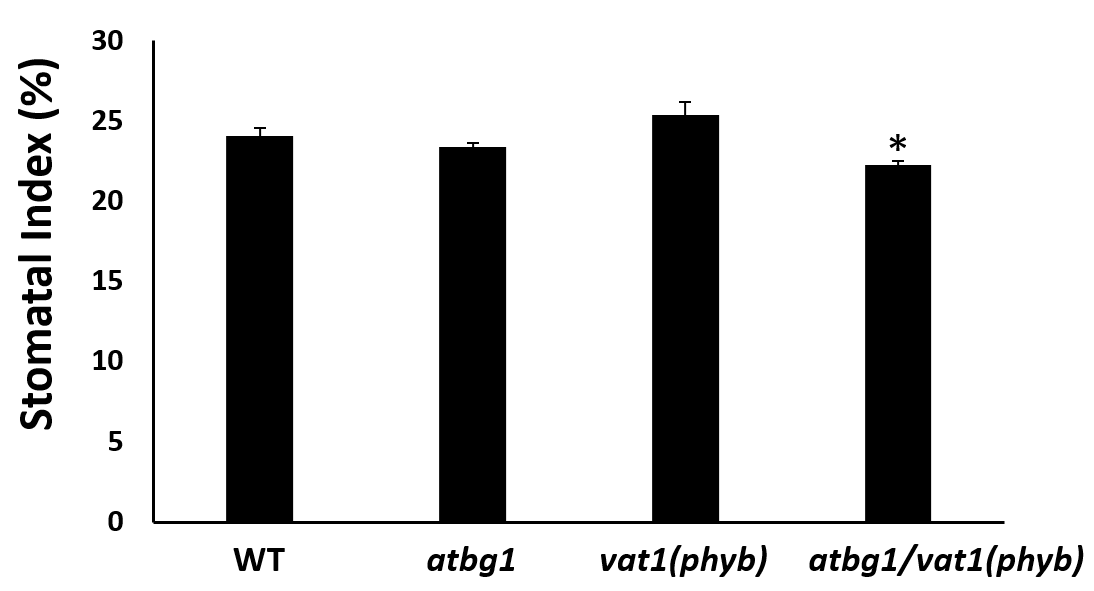


**S1 Fig. Stomatal density and index in the four lines in five-day-old cotyledons.**

Stomatal density: With 0.1<pvalue<0.4, no statistically significant difference between the lines is detected. Error bars represent the standard error (n=3).

Stomatal index: Only the *atbg1/vat1(phyb)* double mutant shows a very modest but statistically significant difference (*P=0.03) compared to WT. Error bars represent the standard error (n=3).
