# Supplementary material for: ABA-glucose ester hydrolyzing enzyme ATBG1 and PHYB antagonistically regulate stomatal development
Source: PLoS One. 2019 Jun 24;14(6):e0218605. doi: 10.1371/journal.pone.0218605 (PMC6590796; doi:10.1371/journal.pone.0218605)
Supplement: S1 Table — (DOCX) [file pone.0218605.s003.docx]

**S1A Table: Primers used for the mapping and distributed over the five chromosomes of *Arabidopsis thaliana*.***

| Name | Forward Primer | Reverse Primer |
| --- | --- | --- |
| nga163 | GCCTAAACCAAGGCACAGAAG | TCATCAGTATTCGACCCAAG |
| F20D23 | TTATGCCAACTCATGTGGAAAG | TGTCAAAGCGTCTGGTTCTG |
| F3I6 | AGATGGAAGAGGAGGAGATGG | TGCATGTATATGATGAGCGAGAG |
| F7P12 | TCGAGGATATGTTTCGTGTTTG | ACAGTTTTGATGCATTGTGTGAG |
| ciw1 | ACATTTTCTCAATCCTTACTC | GAGAGCTTCTTTATTTGTGAT |
| F23H11 | GATATGGGAGTAAGTATGAAATCGG | TTCGTCCGGGTAAAAGTCAAG |
| F20P5 | GATACGTTCAAAATTAGGGACTTC | TGTATTTTGCTAATTGAGGTTATGG |
| F18P14 | ATTCCCGCAATTTATTTTGTTC | GTTTGATGGCAGATTTGTTTTC |
| ciw3 | GGAAACTCAATGAAATCCACTT | GTGAACTTGTTGTGAGCTTTGA |
| F26B6 | CTCTATCTGCCCACGAACAAG | GCCATTGCAAAAGAACATCAG |
| F16P2 | CAGCAATCAAATAACGTGGTG | CTCTCTTCTTTCTTCGCCATTAG |
| F2H17 | ATTGCATACCACGCAGTTCAC | CCATTTTGCCCTTTCCTTCTAC |
| AthBIO2b | TGACCTCCTCTTCCATGGAG | TTAACAGAAACCCAAAGCTTTC |
| nga172 | AGCTGCTTCCTTATAGCGTCC | CCATCCGAATGCCATTGTTC |
| nga162 | CATGCAATTTGCATCTGAGG | CTCTGTCACTCTTTTCCTCTGG |
| MSA6 | TTGGAGGTGCTCTTAGGTTC | GGGCTTTTCACATACGCTTTC |
| N7N14 | CAATACACTTTATCCAGATGCTG | GGGATTTGTTGATTGAAAAAGGAC |
| T6H20 | CGGCTGAAACTTGGAAGGGAC | AGGAAGAACGTGTGATTGTG |
| K27K19 | TGCTTTTGAAGAGATGGTTATTAGG | CCCCATTTCACTTATCATTGG |
| ciw5 | GGTTAAAAATTAGGGTTACGA | AGATTTACGTGGAAGCAAT |
| F14G16 | ACAAACCGATCAGCATTCAAG | GCCTTTGTCACGGATTCAAC |
| T26M18 | CAATTAGCGGAGGCCACTTC | GGGCAAAAGCTTCCAGTAC |
| F28A21 | GCATCATCATTCATCACCAAC | TGTGAAGTGTTTGTCTTTGTG |
| F26K10 | AGAGAGCACGATGCCTGATAG | AATGCTTCAGCGATTGAGAAC |
| F23E13 | TGACCGTTGAAAGTGTTGTTG | GCCCGAGAAGCCTGATAG |
| MHF15 | CTCCTCCTTTAATTTTCTCTCTGTG | AGTTCCAGCTTTGGACTTCTTC |
| nga151a | ATCTCATACTGACCCATATGTTCC | ATTGTACAGTCTAAAAGCGAGAG |
| ciw8a | TACTAGTGAAACCTTTCTCAG | TTTTATGTTTTCTTCAATCAGTTAG |
| nga76 | GGAGAAAATGTCACTCTCCAC | AGGCATGGGAGACATTTACG |
| ciw9 | CAGACGTATCAAATGACAAATG | GACTACTGCTCAAACTATTCGG |
| MNC17 | GTACCGGATCTGTGTTGTGAAG | GTGCTCAAGGAAATGGGATAG |

*From Berendzen et al. (2005).

| Name | Forward Primer | Reverse Primer |
| --- | --- | --- |
| PLS2 | TACGCGAATTATTTTTAGGAGA | AATTTATTTTGAGTCGGATGC |
| PLS3 | TAGTCGTTTCTCTGGTTGTAG | TTGCCTGTCGATGTAGATTTGT |
| NGA361 | ACATATCAATATATTAAAGTAGC | AAAGAGATGAGAATTTGGAC |
| F4P9 | TGGTCCATACCCATTTCATAAC | ATGAATTTTCATTCTACTGTTTTG |
| PLS9 | GAAATTACGCCGAAAGGTC | CGTCACGAGAGGCACATC |
| T20F6 | CGTTCGAAACTGAATTAGCTG | ACCATCTTTGTTGAGCCCT |

**S1B Table: Primers used for the mapping and located on chromosome II.**
