# Supplementary material for: ABA-glucose ester hydrolyzing enzyme ATBG1 and PHYB antagonistically regulate stomatal development
Source: PLoS One. 2019 Jun 24;14(6):e0218605. doi: 10.1371/journal.pone.0218605 (PMC6590796; doi:10.1371/journal.pone.0218605)
Supplement: S2 Table — (DOCX) [file pone.0218605.s004.docx]

**S2 Table**: **List of qPCR primers**

| Name | Forward Primer | Reverse Primer |
| --- | --- | --- |
| BASL | CGCCGGAGACTAAAGGGACAGA | CCCGCTAGATTTATCAGAGGCATCA |
| COP1 | GATGCGCTGAGTGGGTCAGATTC | GACGCCGCTTTTGGAGGTAACA |
| EPF1 | ATTGAAGCGTCAGAGGAGGA | TGAGCAATCTGGCAACCTAGA |
| EPF2 | CGCGTGTTCTTTGGTCGTTAACTC | ACCCCTCCGTTTTTGCTTATTTCC |
| EPFL9/STOMAGEN | CAAGCCTCAAGACCTCGTTCTATCG | CTGCTCTGCACTTGTATCTGCATCC |
| ER/ERECTA | CACTTCACGGCTCACTGAGAAATCC | CCCCGTCTTTGACATTATCAGATGG |
| ERL1 | CCTATCCCACCGATACTTGGCAATC | TGGTATGGGCCCTACTAAACGGTTG |
| ERL2 | TCAAAGCAGCAGAAACCAGTCTTGA | TGGTATGGGCCCTACTAAACGGTTG |
| FAMA | GCCTGGCTCCTACGTTCAAAGG | CATTTCCCGTAATAATGAGCGGTTG |
| FLP | CCGCGAAAATCCGAGAATGAAACTC | ATGCCTCGGCCCTTCCATAGTTAC |
| GSL8 | CAATTGGGGTTTTCCCTGAAGTACG | TGGGACTGTGCATTTGAAAGTGTG |
| MPK3 | GCACACCGACAGAATCTGATCTCG | CATCATTCGGGTGGTGCAATTTAGC |
| MPK6 | CCTTATCCTCGCCAATCCATCACTG | TGGGCCAATGCGTCTAAAACTGT |
| MUTE | TGAACGAGCATCTTAAATCCCTTCG | TGGCGGCTCCTAAACTGGATGG |
| PIF4 | CCAGATCATCTCCGACCGGTTTG | CTAGTGGTCCAAACGAGAACCGT |
| POLAR | GCAGAACTGCCGCCTGAATAAGA | CTCCATTTGCATTCGCAGGTTTGT |
| SCD1 | TCAACGCTGGTGAAAATTGTTTAGG | GCCACCGCTACATATCCGTTTCC |
| SCRM/ICE1 | AGCTTCCATCCGTTGACACCTACA | AGCTTGCTGAACATCCAATCCAAGA |
| SCRM2 | TTTGTGCATGTCGAAAAGAGAATCG | TGGTGGTGGGTTATTGAACCAGTC |
| SPCH | CCGTTTTGCGTTCTCTTATGCCTGG | TTCGGCGTAGGTTTTACGTTGTTTC |
| TMM | AGCTGAGGCTCAACGATAACA | CCTCAGCTTTCCCCTCATCCT |
| YDA | GCCGATGGAAAGGCCTATTGTGA | TTGGTTGCATCTTCCGAGTCTAAGC |
| TUB9 | CGTTCACCAGCTCGTTGAGAATG | TGGAGTCGTGGGAAAGGAATAAGG |
|  |  |  |
|  |  |  |
|  |  |  |
