# Supplementary material for: ABA-glucose ester hydrolyzing enzyme ATBG1 and PHYB antagonistically regulate stomatal development
Source: PLoS One. 2019 Jun 24;14(6):e0218605. doi: 10.1371/journal.pone.0218605 (PMC6590796; doi:10.1371/journal.pone.0218605)
Supplement: S3 Table — (DOCX) [file pone.0218605.s005.docx]

**S3 Table:** Stomatal development genes tested by RT-qPCR

| Gene Name | AGI | Symbol | Gene Description |  |
| --- | --- | --- | --- | --- |
| Differentiation | | | | |
| SPEECHLESS | At5g53210 | SPCH | bHLH protein |  |
| MUTE | At3g06120 | MUTE | bHLH protein |  |
| FAMA | At3g24140 | FAMA | bHLH protein |  |
| SCREAM/ICE1 | At3g26744 | SCREAM | bHLH protein |  |
| SCREAM2 | At1g12860 | SCREAM2 | bHLH protein |  |
| FOUR LIPS | At1g14350 | FLP | R2R3 MYB protein |  |
| MYB88 | At2g02820 | MYB88 | R2R3 MYB protein |  |
| Spacing and Patterning | | | | |
| YODA | At1g63700 | YDA | MAPKKK |  |
| MPK3 | At3g45640 | MPK3 | MAPKKK |  |
| MPK6 | At2g43790 | MPK6 | MAPKKK |  |
| ERECTA | At2g26330 | ER | Leucine-rich repeat receptor kinase |  |
| ERECTA-LIKE1 | At5g62230 | ERL1 | Leucine-rich repeat receptor kinase |  |
| ERECTA-LIKE2 | At5g07180 | ERL2 | Leucine-rich repeat receptor kinase |  |
| TOO MANY MOUTHS | At1g80080 | TMM | Leucine-rich repeat receptor |  |
| EPIDERMAL PATTERNING FACTOR1 | At2g20875 | EPF1 | Cysteine-rich secreted peptide |  |
| EPIDERMAL PATTERNING FACTOR2 | At1g34245 | EPF2 | Cysteine-rich secreted peptide |  |
| STOMAGEN/  EPF-LIKE9 | At4g12970 | EPFL9 | Cysteine-rich secreted peptide |  |
| Polarity and Division Asymmetry | | | | |
| BREAKING OF ASYMMETRY IN THE STOMATAL LINEANAGE | At5g60880 | BASL | No homology |  |
| POLAR LOCALIZATION DURING ASYMMETRIC DIVISION AND REDISTRIBUTION | AT4g31805 | POLAR | No homology |  |
| Cell Division | | | | |
| STOMATAL CYTOKINESIS DEFECTIVE 1 | At1g49040 | SCD1 | DENN-WD40 protein |  |
| GLUCAN SYNTHASE-LIKE 8 | At2g36850 | GSL8 | Callose synthase |  |
| Hormone and Environmental Signaling | | | | |
| PHYTOCHROME INTERACTING FACTOR 4 | At2g43010 | PIF4 | bHLH |  |
| CONSTITUTIVE PHOTOMORPHOGENIC 1 | At2g32950 | COP1 | Ubiquitin Ligase |  |
|  |  |  |  |  |
|  |  |  |  |  |
